# The 2017 global point prevalence survey of antimicrobial consumption and resistance in Canadian hospitals

**DOI:** 10.1186/s13756-020-00758-x

**Published:** 2020-07-11

**Authors:** Charles Frenette, David Sperlea, Greg J. German, Kevin Afra, Jennifer Boswell, Sandra Chang, Herman Goossens, Jennifer Grant, Marie-Astrid Lefebvre, Allison McGeer, Dominic Mertz, Michelle Science, Ann Versporten, Daniel J. G. Thirion

**Affiliations:** 1 McGill University Health Center, 1001 Decarie Blvd, Montreal, Quebec H4A 3J1 Canada; 2 Faculty of Pharmacy, Université de Montréal, 2940 Chemin de Polytechnique, Montreal, Quebec H3T 1J4 Canada; 3 Health PEI, 16 Garfield St, Charlottetown, Prince Edward Island C1A 6A5 Canada; 4 Fraser Health, 2733 Heather Street, Vancouver, British Columbia V5Z VGH Canada; 5 Richmond Hospital, 7000 Richmond Hwy, Richmond, British Columbia V6X 1A2 Canada; 6 Laboratory of Medical Microbiology, Vaccine & Infectious Disease Institute, University of Antwerp, 2610 Wilrijk, Antwerp, Belgium; 7 Vancouver General Hospital, 899 W12th Ave, Vancouver, British Columbia V5Z 1M9 Canada; 8 Montreal Children Hospital, 1001 Decarie Blvd, Montreal, Quebec, H4A 3J1 Canada; 9 Mount Sinai Hospital, 600 University Ave, Toronto, Ontario M5G 1X5 Canada; 10 Hamilton Health Sciences, 711 Concession Street, Hamilton, Ontario L8V 1C3 Canada; 11 The Hospital for Sick Children, 555 University Ave, Toronto, Ontario M5G 1X8 Canada

**Keywords:** Benchmarking, Antibiotic use, Antimicrobial indication, Antibiotic stewardship, Antibiotic resistance

## Abstract

**Background:**

Patient-level surveillance (indication, appropriate choice, dosing, route, duration) of antimicrobial use in Canadian hospitals is needed to reduce antimicrobial overuse and misuse. Patient-level surveillance has not been performed on a national level in Canada. The Global Point Prevalence Survey of Antimicrobial Consumption and Resistance (Global-PPS) is an international collaborative to monitor antimicrobial use and resistance in hospitals worldwide. Global-PPS locally documents on a single day patient-level antimicrobial prescribing practices. This article presents the results of the 2017 Global-PPS in Canadian hospitals with established antimicrobial stewardship programs.

**Methods:**

Hospitals part of the Canadian Nosocomial Infection Surveillance Program were invited to participate. Surveys could be performed any time in the 2017 calendar year. All in-patient wards in each hospital were surveyed by a physician, pharmacist or nurse with infectious disease training.

**Results:**

Fourteen Canadian hospitals participated in the survey. Of 4118 patients, 1400 patients (34.0%) received a total of 2041 antimicrobials. Overall, 73.1% (*n* = 1493) of antimicrobials were for therapeutic use, 14.2% (*n* = 288) were for medical prophylaxis, 8.3% (*n* = 170) were for surgical prophylaxis, 1.8% (*n* = 37) were for other reasons, and 0.2% (*n* = 3) were used as prokinetic agents. Only 2.5% (*n* = 50) were for unknown reasons. For antimicrobials for therapeutic use, 29.9% of patients were treated for lower respiratory tract (343/1147), 10.5% for intra-abdominal (120/1147), 9.3% for skin and soft tissue (107/1147) and 7.5% for gastro-intestinal (86/1147) infections.

**Conclusions:**

Standardized methodology amongst Global-PPSs allows the comparison of our results to the 2015 Global-PPS. The prevalence of antimicrobial use on medical, surgical, and intensive care wards are similar to those previously observed in North America. Indication of antimicrobials has not been previously reported on such a large scale in Canadian hospitals. This report serves as a comparison for further point prevalence surveys that are currently underway. It will be used for identifying opportunities and benchmarking in antibiotic stewardship.

## Background

Antimicrobial resistance (AMR) is one of the biggest threats to healthcare [1]. Although the evolution of AMR is complex and AMR’s future burden is unpredictable, AMR increases mortality, morbidity and healthcare costs [2]. Given that antimicrobial use (AMU) accelerates the development of AMR, antimicrobial overuse and misuse must be decreased to preserve its effectiveness. A global response is imperative to ensure prudent AMU since AMR is commutable between countries. The World Health Organization adopted the *Global Action Plan on Antimicrobial Resistance* to guide international efforts for effective prevention and treatment of infectious diseases [3].

Canada implemented the *Framework for Action* on AMR and AMU in 2017 to strengthen its combat against AMR and complement the *Global Action Plan* [4]. Surveillance of AMU is a necessary step to monitor trends and identify areas of concern and is a core component of the *Framework for Action*. As part of the *Framework*, the Canadian Nosocomial Infection Surveillance Program (CNISP) monitors AMU in participating Canadian hospitals. Quantitative AMU measured by daily defined doses (DDDs) in CNISP hospitals has remained overall stable since 2009 but with significant variation between antimicrobial classes [4]. However, population-level AMU surveillance through DDDs lacks patient-level information, and qualitative surveillance (indication, appropriate choice, dosing, route, duration) is required to interpret quantitative aspects and to guide complete antimicrobial stewardship interventions [5]. Patient-level surveillance of AMU in Canadian hospitals is needed to reduce antimicrobial overuse and misuse. The CNISP performed patient-level AMU surveillance in the past through point prevalence surveys (PPS) of healthcare-associated infections (HAIs) [6]. However, CNISP hospitals are large tertiary care university-affiliated centers, which may overestimate AMU [6]. Patient-level AMU surveillance has not been performed on a national level in Canada.

The Global Point Prevalence Survey of Antimicrobial Consumption and Resistance (Global-PPS) is an international collaborative created in 2014 to monitor antimicrobial use and resistance in hospitals worldwide. Global-PPS locally documents on a single day patient-level antimicrobial prescribing practices. The advantage of the Global-PPS’s standardized surveillance method is that it is adapted to all types of hospitals and allows data comparison locally, nationally and internationally. Global-PPS identifies areas of improvement and, through repeat surveys, the impact of interventions can be measured. This article presents the results of the 2017 Global-PPS in Canadian hospitals with established antimicrobial stewardship programs.

## Methods

### Objective and design

The objective of this cross-sectional study was to evaluate antimicrobial use and resistance in Canadian hospitals with an established antimicrobial stewardship program. The primary outcome was to measure antimicrobial prescribing rates, antimicrobial indications and agent selection in medical, surgical and intensive care wards. The secondary outcome was to measure resistance rates.

### Setting and participants

Hospitals part of the CNISP (67 hospitals) were invited to participate in the 2017 Global-PPS. Surveys were performed between February and July 2017; two hospitals performed the study beginning November 2017. All in-patient wards in each hospital were surveyed. Each ward was surveyed once on a single day, but different wards could be surveyed on separate days, with the exception that wards were not surveyed on a weekend day or a holiday. Surgical wards were in addition not surveyed on a day following a weekend day or holiday in order to capture information on the duration of surgical prophylaxis (SP).

On the day of the survey, detailed data was collected for all admitted inpatients receiving an antimicrobial as of 0800 h. A patient was considered on antimicrobial therapy if the agent was one of the following: systemic antibiotics, antibiotics used as intestinal anti-infectives, systemic antimycotics and antifungals, antituberculosis agents, nitroimidazole derivatives and antiprotozoals used as antibacterial agents, neuraminidase inhibitors and antimalarials. The included routes of administration were parenteral (which also includes subcutaneous, intramuscular, intraventricular, intraperitoneal and other specific routes of administration; see Frequently Asked Questions on Global-PPS’s website), oral, rectal and inhalation [7].

Numerator data included patients on antimicrobial therapy. A patient admitted or who was prescribed an antimicrobial after 0800 h was excluded. Denominator data included all patients hospitalized on the ward at 0800 h the day of the survey. Day hospitalizations and outpatients were excluded from the numerator and denominator.

Participation in the Global-PPS was deemed a quality improvement project through the CNISP and therefore express approval from Ethics Board Comities was not needed.

### Data collection

A physician, pharmacist or nurse with infectious disease training performed the survey. An administrator per site provided oversight to ensure survey completion. The necessary detailed information was retrieved from medical records and not discussed with the ward staff nor was direct feedback provided to enhance objective data collection. The Global-PPS utilizes a uniform standardized surveillance method for all hospitals. Data specific to wards included ward type and specialty, number of patients hospitalized on the ward and number of available beds at 0800 h on the day of the PPS.

Wards were categorized by type as follows: medicine, surgery and intensive care. Adult wards were further categorized by specialty as follows: adult medical ward (AMW), haematology-oncology-AMW, transplant-AMW, pneumology-AMW, adult surgical ward and adult intensive care unit. Pediatric wards were categorized by specialty as follows: pediatric medical ward (PMW), haematology-oncology-PMW, transplant (solid/bone marrow transplant)-PMW, pediatric surgical ward and pediatric intensive care unit. Neonatal wards were categorized by specialty as follows: neonatal medical ward and neonatal intensive care unit.

Data collected for each patient on antimicrobial therapy included the following: age, weight, gender and antimicrobial agent. For each antimicrobial received, the following information was collected: dose, route (oral, parenteral, rectal, inhalation), diagnosis, indication, and a set of quality indicators such as diagnosis documented in the chart at the start of the antimicrobial (yes/no), local guideline compliance (yes/no/not assessable/no information), stop/review date documented (yes/no) and whether therapy was empirical or targeted. The physician’s diagnosis was recorded based on standardized categories (protocol available at www.global-pps.com/) [7]. Type of indication was categorized based on standardized definitions and included: community-acquired infection (CAI), HAI, SP as one dose, one day or more than one day, medical prophylaxis (MP), other and unknown. If therapy was targeted, the targeted organism was recorded.

### Data analysis

Antimicrobial consumption data is presented in terms of proportions. Prevalence of antimicrobial prescribing is presented as the proportion of patients on at least one antimicrobial compared to the number of inpatients on the ward. A patient on single or multiple antimicrobials had the same weight in the numerator. Dose differences between patients for the same antimicrobial were not analyzed.

## Results

Fourteen Canadian hospitals participated in the 2017 Global-PPS and data from all hospitals was included in the study. Ten hospitals were university-affiliated centers. Three hospitals were from Western Canada, nine from Central Canada and two from the Atlantic Provinces. Two hospitals were primary care centers (179 patients surveyed, 4.4% of patients surveyed), 3 were secondary care centers (1110 patients surveyed, 26.7% of patients) and 7 were tertiary/specialized care centers (2839 patients surveyed, 68.9% of patients). Two tertiary care centers were exclusively pediatric centers. Overall, 237 units and 4118 patients (3447 adults, 410 pediatric patients, and 261 neonates) were included in the survey. The average age of adults was 64.5 ± 18.0 (standard deviation) years old and 6.3 ± 5.7 (standard deviation) years old for pediatric patients (age of neonates was not recorded due to legal/privacy reasons). The percentage of male patients was 54.7%, 52.2% and 46.7% in adult, pediatric and neonatal patients, respectively.

### Antimicrobial prevalence

Of 4118 admitted inpatients, 1400 patients (34.0%) received a total of 2041 antimicrobials. 22.9% of patients in primary care centers received antimicrobials, 29.4% in secondary care centers, and 36.4% in tertiary/specialized care centers. 34.4% of adults, 44.4% of pediatric patients and 11.9% of neonates received at least one antimicrobial (Table 1). In adult wards, antimicrobial prevalence was highest in bone marrow/solid organ transplant wards (78.0%) and lowest in adult medical wards (28.4%). Out of 2041 antimicrobial prescriptions, 73.1% (*n* = 1493) were for therapeutic use, 14.2% (*n* = 288) were for MP, 8.3% (*n* = 170) were for SP, 1.8% (*n* = 37) were for other reasons, and 0.2% (*n* = 3) were used as prokinetic agents. Only 2.5% (*n* = 50) were for unknown reasons.

**Table 1:** Overall Antimicrobial Prevalence by Ward Type in Adult, Pediatric and Neonatal Patients

**Adult**	**Overall**	**AMW**	**HO-AMW**	**T-AMW**	**P-AMW**	**ASW**	**AICU**
Number of patients on ward, N	3447	1947	47	50	54	944	405
Number of patients receiving antimicrobials, N (%)	1187 (34.4)	553 (28.4)	17 (36.2)	39 (78.0)	29 (53.7)	364 (38.6)	185 (45.7)
Number of antimicrobials received, N	1685	764	29	97	59	475	261
**Pediatric and Neonatal**	**Overall**	**PMW and GNMW**	**HO-PMW**	**T-PMW**		**PSW**	**PICU and NICU**
Number of patients on ward, N	671	278	37	40		105	211
Number of patients receiving antimicrobials, N (%)	213 (31.7)	66 (23.7)	32 (86.5)	23 (57.5)		34 (32.4)	58 (27.5)
Number of antimicrobials received, N	356	88	70	49		46	103

*Abbreviations*: *AICU* adult intensive care unit, *AMW* adult medical ward, *ASW* adult surgical ward, *GNMW* general neonatal medical ward, *HO-AMW* hematology-oncology AMW, *NICU* neonatal intensive care unit, *P-AMW* pneumology-AMW, *PICU* pediatric intensive care unit, *PMW* pediatric medical ward, *PSW* pediatric surgical ward, *T-AMW* transplant-AMW

### Therapeutic use

**Fig. 1:**
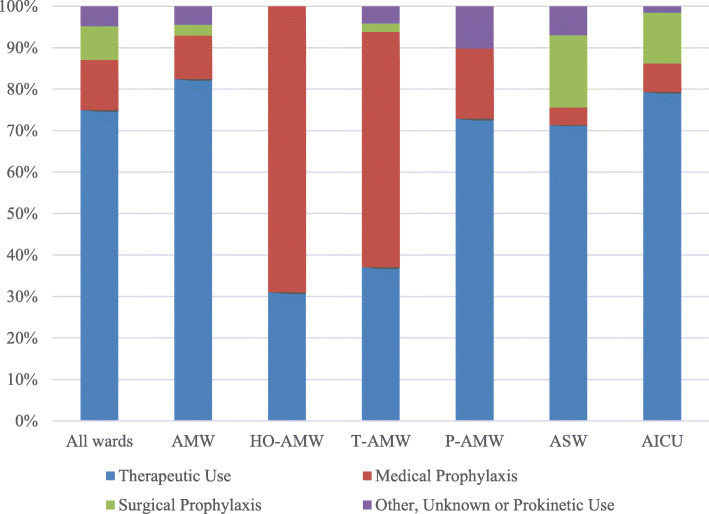
Antimicrobial Use (%) by Ward Type for Adults. Abbreviations: AICU, adult intensive care unit; AMW, adult medical ward; ASW, adult surgical ward; HO-AMW, hematology-oncology AMW; P-AMW, pneumology-AMW; T-AMW, transplant-AMW

**Fig. 2:**
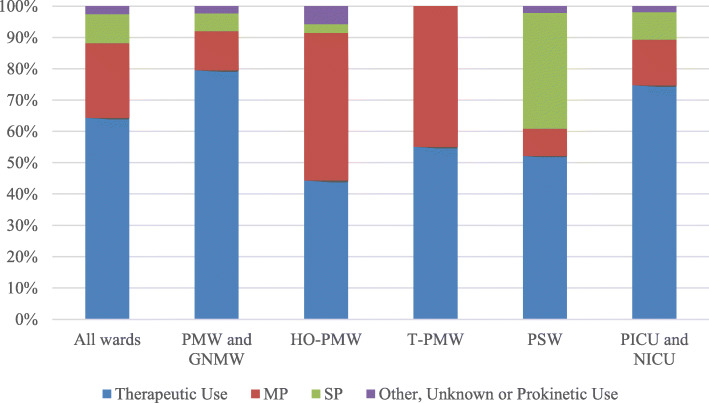
Antimicrobial Use (%) by Ward Type for Pediatric and Neonatal Wards. Abbreviations: GNMW, general neonatal medical ward; HO-PMW, hematology-oncology PMW; NICU, neonatal intensive care unit; PICU, pediatric intensive care unit; PMW, pediatric medical ward; PSW, pediatric surgical ward; T-PMW, transplant-PMW

Therapeutic use accounted for the majority of antimicrobial prescriptions (75.0% in adults, 61.5% in pediatric patients, and 78.3% in neonates; Figs. 1 and 2). Overall, 29.9% of patients were treated for lower respiratory tract (343/1147), 10.5% for intra-abdominal (120/1147), 9.3% for skin and soft tissue (107/1147) and 7.5% for gastro-intestinal (86/1147) infections (Supplementary Material Table S1). In adults, 53.5% (676/1263) of antimicrobials were for CAIs, 45.5% (575/1263) were for HAIs, and 1.0% (13/1263) of episodes treated were of unknown origin. In pediatric patients, 62.1% (113/182) and 37.9% (69/182) of antimicrobials were for CAIs and HAIs, respectively. In neonates, 36.2% (17/47) and 63.8% (30/47) of antimicrobials were for CAIs and HAIs, respectively. Empirical treatment accounted for 59.7% (890/1492) of all antimicrobials for therapeutic use and targeted treatment for 40.4% (603/1492). Of 809 antimicrobials for CAIs, 526 (65.0%) were empirical treatment and 283 (35.0%) were targeted treatment. Of the 678 antimicrobials for HAIs, 358 (52.8%) were empirical treatment and 320 (47.2%) were targeted treatment.

### Medical prophylaxis

Antifungals were the most commonly prescribed antimicrobials for MP (33.0%, 95/288; Supplementary Material Table S2). Combinations of sulfonamides and trimethoprim were the second most prescribed (28.1%, 81/288). General MP (55.5%, 160/288), followed by respiratory tract prophylaxis (22.2%, 64/288), were the most common indications for antimicrobial use.

### Surgical prophylaxis

Cefazolin accounted for the majority (55.3%) of surgical antimicrobial prophylaxis prescriptions. Overall, 11% of patients received a single dose of SP, 38% received prophylaxis for a duration of 1 day and 52% for more than 1 day. Supplementary Material Table S3 presents antimicrobial prevalence by SP site in adult, pediatric, and neonatal wards.

### Antimicrobial class prevalence

**Fig. 3:**
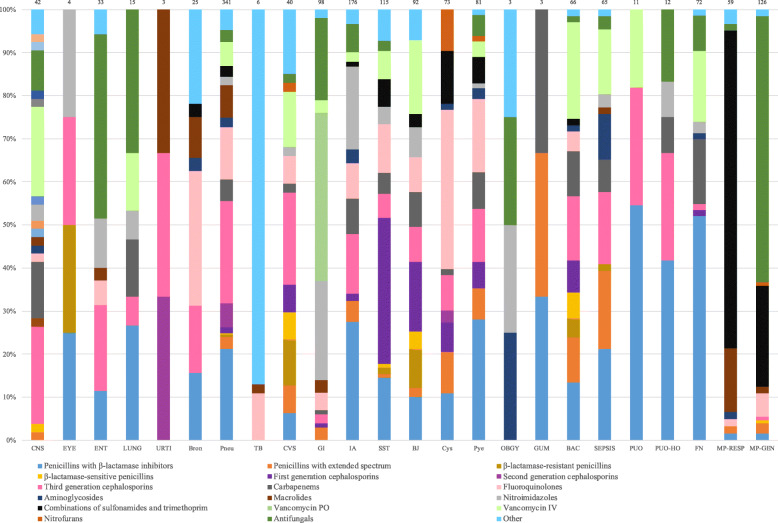
Antimicrobial Class Use by Indication. All indications are for therapeutic use except for general prophylaxis (MP-GEN) and respiratory prophylaxis (MP-RESP)^a^. ^a^ See the Global-PPS’s protocol for abbreviations of indications

Antibiotics accounted for 86.3% of antimicrobials prescribed (1762/2041; Tables 2 and 3). Penicillins with β-lactamase inhibitors (17.4%, 306/1762), 3rd generation cephalosporins (12.5%, 221/1762) and 1st generation cephalosporins (11.0%, 194/1762; Tables 2 and 3) were the most common antibiotics prescribed. Out of 93 metronidazole prescriptions for therapeutic use, 15 were for healthcare-associated *Clostridioides difficile*-associated diarrhea (16.1%). For treatment of pneumonia, the combination of a β-lactam and macrolide or fluoroquinolone monotherapy accounted for a modest proportion (Fig. 3). Fluoroquinolones were the most commonly used antibiotic for treatment of cystitis. Together, nitrofurans and combinations of sulfonamides and trimethoprim accounted for <25% of antibiotics for the treatment of cystitis.

**Table 2:** Antimicrobial Prevalence by Class in Adult Wards

Adult	Overall^a,b^	AWM	HO-AMW	T-AMW	P-AMW	ASW	AICU
**Total number of antimicrobials**	1685	764	29	97	59	475	261
**Antibiotics, N**	1454 (86.3)	665	14	50	44	439	242
**Antifungals, N**	164 (9.7)	65	7	36	8	29	19
**Antivirals, N**	18 (1.1)	2	6	8	0	2	0
**Antituberculosis agents, N**	30 (1.8)	21	0	0	7	2	0
**Other, N**	19 (1.1)	11	2	3	0	3	0
**Antibiotics by Class, N**							
Penicillins with β-lactamase inhibitors	275 (18.9)	113	5	20	9	86	42
Penicillins with extended spectrum	36 (2.5)	26				9	1
β-lactamase-resistant penicillins	19 (1.3)	12				3	4
β-lactamase-sensitive penicillins	15 (1.0)	11				3	1
1st generation cephalosporins	164 (11.3)	60			2	79	23
2nd generation cephalosporins	18 (1.2)	15				1	2
3rd generation cephalosporins	169 (11.6)	83		2	4	46	34
5th generation cephalosporins	1 (0.1)	1					
Carbapenems	85 (5.8)	31	1	4	3	23	23
Fluoroquinolones	165 (11.3)	87	2	5	3	50	18
Aminoglycosides	13 (0.9)	3			3	6	1
Macrolides	55 (3.8)	34	1	2	7	6	5
Tetracyclines	30 (2.1)	19	1		2	4	4
Clindamycin	10 (0.7)	4				3	3
Metronidazole	101 (6.9)	34		2	1	54	10
Combinations of sulfonamides and trimethoprim	81 (5.6)	35	4	7	7	14	14
Linezolid	6 (0.4)	2			1	1	2
Vancomycin PO	40 (2.8)	27		2		7	4
Vancomycin IV	112 (7.7)	38		5		27	42
Nitrofurantoin	12 (0.8)	4				8	
Daptomycin	15 (1.0)	7		1		5	2
Tigecycline	1 (0.1)						1
Rifamycins	29 (2.0)	18			2	4	5
Fosfomycin	2 (0.1)	1					1

^a^ Numbers in parentheses represent a percentage

^b^ The denominator to calculate percentages for each antibiotic subclass is the total number of antibiotics

*Abbreviations*: *AICU* adult intensive care unit, *AMW* adult medical ward, *ASW* adult surgical ward, *HO-AMW* hematology-oncology AMW, *P-AMW* pneumology-AMW, *T-AMW* transplant-AMW

**Table 3:** Antimicrobial Prevalence by Class in Pediatric and Neonatal Wards

Pediatric and Neonatal	Overall^a,b^		PMW and GNMW	HO-PMW	T-PMW	PSW	PICU and NICU
**Total number of antimicrobials**	356		88	70	49	46	103
**Antibiotics, N**	308 (86.5)		82	58	26	44	98
**Antifungals, N**	34 (9.6)		5	10	14	1	4
**Antivirals, N**	13 (3.7)		1	2	9	0	1
**Antituberculosis agents, N**	0 (0)		0	0	0	0	0
**Other, N**	1 (0.3)		0	0	0	1	0
**Antibiotics by Class, N**							
Penicillins with β-lactamase inhibitors	31 (10.1)		8	8	9	3	3
Penicillins with extended spectrum	44 (14.3)		12	3	1	4	24
β-lactamase-resistant penicillins	4 (1.3)		2				2
β-lactamase-sensitive penicillins	1 (0.3)						1
1st generation cephalosporins	30 (9.7)		12	2	2	8	6
2nd generation cephalosporins	6 (1.9)		4			1	1
3rd generation cephalosporins	52 (16.9)		19	4	4	5	20
Carbapenems	8 (2.6)		1	2		1	4
Fluoroquinolones	10 (3.2)		3	5	2	0	0
Aminoglycosides	29 (9.4)		3	2		5	19
Macrolides	4 (1.3)		2			1	1
Tetracyclines	1 (0.3)		1				
Clindamycin	7 (2.3)			1		6	
Metronidazole	14 (4.5)		3	3		5	3
Combinations of sulfonamides and trimethoprim	40 (13.0)		6	22	3	3	6
Vancomycin PO	3 (1.0)		1	1	1		
Vancomycin IV	23 (7.5)		5	4	4	2	8
Rifamycins	1 (0.3)			1			

^a^ Numbers in parentheses represent a percentage

^b^ The denominator to calculate percentages for each antibiotic subclass is the total number of antibiotics

*Abbreviations*: *GNMW* general neonatal medical ward, *HO-PMW* hematology-oncology PMW, *NICU* neonatal intensive care unit, *PICU* pediatric intensive care unit, *PMW* pediatric medical ward, *PSW* pediatric surgical ward, *T-PMW* transplant-PMW

### Antibiotic stewardship

A diagnosis/indication was documented in the patient’s file at the start of 84% of antimicrobials (1720/2041). 62% of antimicrobials had a stop/review date documented in the patient’s file. Local guidelines were present to guide 79% of antimicrobial prescriptions and 82% of prescriptions complied with the recommended antimicrobial choice.

### Antimicrobial resistance

The most frequent multi-drug resistant organisms treated were methicillin-resistant *Staphylococcus aureus* (2.7%, 29/1088 patients treated), bacteria producing extended-spectrum beta-lactamases (ESBLs) (1.8%, 20/1088) and 3rd generation cephalosporin resistant organisms (non-ESBL or ESBL status unknown) (1.0%, 10/1088; Table 4).

**Table 4:** Antimicrobial Resistance Rates in all Hospitals

Multidrug-resistant organism	Number of patients treated for MDRO	Prevalence of MDRO (%, number of patients treated for MDRO on number of patients receiving antimicrobials for therapeutic use (CAI and HAI))
MRSA	29	2.7
MRCoNS	6	0.6
VRE	10	0.9
ESBL	20	1.8
3-ceph	11	1.0
CRE	0	
ESBL-NF	2	0.2
CR-NF	2	0.2
Other MDRO	19	1.7

*Abbreviations*: *3-ceph* 3rd generation cephalosporin resistant *Enterobacteriaceae* (non-ESBL or ESBL status unknown), *CAI* community-acquired infection, *CRE* carbapenem-resistant *Enterobacteriaceae*, *CR-NF* carbapenem-resistant non-fermenter Gram-negative bacilli, *ESBL* bacteria producing extended-spectrum beta-lactamases, *ESBL-NF* ESBL-producing non-fermenter Gram-negative bacilli, *HAI* healthcare-associated infection, *MDRO* multi-drug resistant organism, *MRCoNS* methicillin-resistant coagulase negative staphylococci, *MRSA* methicillin-resistant *Staphylococcus aureus*, *VRE* Vancomycin-resistant enterococci

## Discussion

The prevalence of antimicrobial use on medical, surgical and intensive care wards are similar to those previously observed in North America in the 2015 Global-PPS [8]. Reported rates in hospitals in the United States are close to 50% in a survey which includes patients that receive an antimicrobial in a 48-h period [9]. Although SP is routinely used for most surgeries, it accounted for only 17.5% of antimicrobial consumption on adult surgical wards with therapeutic use at 71.4%. Our results also demonstrate that significant variation in proportions of antimicrobials for therapeutic use, MP and SP exists between ward type and specialty. In adult medical, surgical and intensive care wards and pediatric medical wards, therapeutic use accounts for more than 70% of consumption whereas MP accounts for the majority in hematology-oncology and transplant wards. Indications for therapeutic use also vary by ward type. Respiratory tract infections account for the majority of infections treated in most wards except for transplant-AMWs, where febrile neutropenia is the main indication. The proportion of patients treated for GI tract, skin and soft tissue, bone and joint and urinary tract infections varies according to ward speciality. Given that antimicrobial prescribing practices differ by ward type and specialty, future studies that report quantitative consumption should present data by ward speciality to appreciate heterogeneity between wards.

The Global-PPS was first performed in 2015 which established the feasibility across a wide range of hospitals in 53 countries. Only 14/67 CNISP hospitals participated in 2017. However, participation is a significant undertaking that is resource intensive, especially if paper charting is present. It requires training personnel to perform the survey and mobilization of a multidisciplinary team on a single day. Although many of the hospitals participated for the first time in 2017, Global-PPS’s protocol was easily implemented. The majority of hospitals performed the survey on the same day.

Standardized methodology amongst Global-PPSs allows the comparison of our results to the 2015 Global-PPS [8]. Lower respiratory tract infection was the most common indication for treatment at similar proportions in both studies. Intra-abdominal, skin and soft tissue and gastro-intestinal infections, respectively, were the next 3 most common indications for treatment in our study. However, urinary tract, skin and soft tissue and intra-abdominal infections were the next 3 most common indications for treatment in North America in the 2015 Global-PPS. Similar to the 2015 Global-PPS, penicillins with β-lactamase inhibitors were the most common prescribed antibiotic, followed by 3rd generation cephalosporins. Also, a significant decrease in fluoroquinolone use is noted in our study relative to PPSs performed in 2002 and 2009 in Canadian hospitals part of the CNISP network [6]. Interestingly, fluoroquinolone purchasing decreased by 43% in Canadian hospitals between 2010 and 2016, whereas penicillin combinations and β-lactamase-sensitive penicillins purchasing increased by 41% [4].

Expressing resistance levels as a prevalence rate should not be compared to the commonly used incidence rates because culture results are not yet available for patients undergoing empiric therapy. Still a prevalence rate might provide a sufficient proxy to estimate current local resistance levels. Another PPS in Canadian hospitals measured a 4.1% prevalence rate for methicillin-resistant *Staphylococcus aureus*, 0.8% for vancomycin-resistant enterococci, 0.8% for extended spectrum beta-lactamase bacteria, and 0% for carbapenem-resistant *Enterobacteriaceae* [10]. However, the prevalence rate was measured according to patients infected and colonized with the organisms, while the current Global-PPS rates are measured for patients who are actively receiving treatment.

The main limitation of PPSs is inherent to the method used, namely the interpretation of single point data. Although day-to-day variations occur, PPSs have moderate correlation with antimicrobial consumption measured in DDD for the month of the PPS but have less correlation when compared to the annual DDD average, which suggests seasonal variation [11]. Additional Global-PPSs performed in the same year will alleviate this bias. Also, Global-PPS relies on voluntary participation and 10/14 hospitals were university-affiliated tertiary/specialized care centers which may overestimate antimicrobial consumption in this study for Canada. Our results support that primary care centers have the lowest overall antimicrobial use prevalence and tertiary/specialized care centers have the highest. A next step will be to analyze Global-PPS results by the 2019 WHO AWaRe (Access, Watch, Reserve) classification list to describe and monitor patterns of inappropriate antimicrobial use in more detail [12].

An indication/diagnosis was documented in 84% of charts. The remaining cases are likely for nonindicated MP; however, this was not investigated, therefore it is difficult to comment on. A missing component of the survey was the validity of the infectious disease diagnosis. The surveyor recorded what the physician intended to treat as recorded in the medical files, which was not based on strict case definitions provided with the Global-PPS protocol. A substantial proportion of inappropriate use is due to inaccurate diagnosis [13]. The goal of the Global-PSS was to measure antimicrobial consumption and not to perform stewardship by intervening on the wards.

## Conclusions

This study presents Canadian hospital antimicrobial point prevalence consumption data for adults and children according to antibiotic classes for indications, ward types and specialties, as well as the prevalence of resistance rates. Indication of antimicrobials has not been previously reported on such a large scale in Canadian hospitals. This report serves as an initial comparison for further PPSs that are currently underway. It will be used to identify opportunities and benchmarking in antimicrobial stewardship.

## Supplementary information

**Additional file 1: Table S1.** Number of Patients Receiving Antimicrobials for Community-Acquired and Hospital-Acquired Infectious Disease Indications. **Table S2.** Antimicrobial Prevalence by Medical Prophylaxis Site for Adult, Pediatric and Neonatal Wards. **Table S3.** Antimicrobial Prevalence by Surgical Prophylaxis Site for Adult, Pediatric and Neonatal Wards.

## Data Availability

Data is available upon reasonable request to the corresponding author.

## Abbreviations

AMR: Antimicrobial resistance
AMU: Antimicrobial utilization
AMW: Adult medical ward
CAI: Community-acquired infection
CNISP: Canadian Nosocomial Infection Surveillance Program
DDD: Daily defined dose
ESBL: Extended spectrum beta-lactamase
Global-PPS: Global Point Prevalence Survey of Antimicrobial Consumption and Resistance
HAI: Healthcare-associated infection
MP: Medical prophylaxis
SP: Surgical prophylaxis
PMW: Pediatric medical ward
PPS: Point prevalence survey
