# Supplementary material for: The 2017 global point prevalence survey of antimicrobial consumption and resistance in Canadian hospitals
Source: Antimicrob Resist Infect Control. 2020 Jul 11;9:104. doi: 10.1186/s13756-020-00758-x (PMC7353732; doi:10.1186/s13756-020-00758-x)
Supplement: Supplementary file 1 — Additional file 1: Table S1. Number of Patients Receiving Antimicrobials for Community-Acquired and Hospital-Acquired Infectious Disease Indications. Table S2. Antimicrobial Prevalence by Medical Prophylaxis Site for Adult, Pediatric and Neonatal Wards. Table S3. Antimicrobial Prevalence by Surgical Prophylaxis Site for Adult, Pediatric and Neonatal Wards. [file 13756_2020_758_MOESM1_ESM.docx]

**Supplementary Material Table S1.** Number of Patients Receiving Antimicrobials for Community-Acquired and Hospital-Acquired Infectious Disease Indications

| **Adult** | **Overall**  **CAI/HAI** | | **AMW**  **CAI/HAI** | | | **HO-AMW**  **CAI/HAI** | | | **T-AMW**  **CAI/HAI** | | | **P-AMW**  **CAI/HAI** | | | **ASW**  **CAI/HAI** | | | **AICU**  **CAI/HAI** | |
| --- | --- | --- | --- | --- | --- | --- | --- | --- | --- | --- | --- | --- | --- | --- | --- | --- | --- | --- | --- |
| Central Nervous System | 9/7 | | | 3/3 | | |  | | |  | | |  | | | 1/3 | | | 5/1 |
| Ear, Nose, Throat | 7/14  (1 UNK) | | | 3/10 | | |  | | |  | | |  | | | 3/2 (1 UNK) | | | 1/2 |
| Respiratory Tract | 180/122 (1 UNK) | | | 110/51 | | | 2/0 | | | 0/2 | | | 17/4 | | | 13/24 | | | 36/41 ( 1 UNK) |
| Cardiovascular | 20/12 | | | 9/7 | | |  | | |  | | |  | | | 6/4 | | | 5/1 |
| GI Tract | 87/93 (1UNK) | | | 31/44 (1 UNK) | | |  | | | 1/0 | | | 6/0 | | | 45/33 | | | 4/16 |
| Skin/Soft Tissue | 50/45 (1 UNK) | | | 28/13 | | |  | | | 1/1 | | |  | | | 19/23 | | | 2/8 (1 UNK) |
| Bone and Joint | 53/17  (1 UNK) | | | 33/6 (1 UNK) | | |  | | |  | | |  | | | 15/11 | | | 5/0 |
| Urinary Tract | 65/75 | | | 44/42 | | | 1/1 | | |  | | | 0/2 | | | 17/28 | | | 3/2 |
| Genitourinary/ Obstetrics | 2/4 | | | 1/4 | | |  | | |  | | |  | | | 1/0 | | |  |
| BAC | 16/37 | | | 12/20 | | | 0/2 | | | 0/2 | | |  | | | 2/7 | | | 2/6 |
| SEPSIS | 9/10 | | | 2/4 | | |  | | |  | | |  | | | 0/4 | | | 7/2 |
| PUO/PUO-HO | 4/5 (3 UNK) | | | 2/2 (1 UNK) | | |  | | | 1/3 (1 UNK) | | |  | | | 1/0 | | | 1 UNK |
| FN | 10/28 (1 UNK) | | | 6/11 (1 UNK) | | | 0/3 | | | 0/14 | | |  | | | 4/0 | | |  |
| **Pediatric and Neonatal** | | **Overall**  **CAI/HAI** | | | **PMW and GNMW**  **CAI/HAI** | | | **HO-PMW**  **CAI/HAI** | | | **T-PMW**  **CAI/HAI** | | | **PSW**  **CAI/HAI** | | | **PICU and NICU**  **CAI/HAI** | | |
| Central Nervous System | | 6/4 | | | 3/0 | | |  | | | 0/1 | | | 1/0 | | | 2/3 | | |
| Eye | | 1/1 | | | 1/1 | | |  | | |  | | |  | | |  | | |
| Ear, Nose, Throat | | 5/2 | | | 1/0 | | | 0/1 | | |  | | | 4/1 | | |  | | |
| Respiratory Tract | | 22/18 | | | 18/0 | | | 0/1 | | | 0/3 | | | 0/2 | | | 4/12 | | |
| Cardiovascular | | 0/1 | | |  | | |  | | |  | | |  | | | 0/1 | | |
| GI Tract | | 15/10 | | | 7/0 | | | 2/3 | | | 1/2 | | | 5/1 | | | 0/4 | | |
| Skin/Soft Tissue | | 7/5 | | | 4/3 | | | 1/1 | | | 1/0 | | | 1/1 | | |  | | |
| Bone and Joint | | 3/3 | | | 2/0 | | | 0/2 | | |  | | | 1/0 | | | 0/1 | | |
| Urinary Tract | | 6/1 | | | 4/0 | | |  | | | 2/0 | | |  | | | 0/1 | | |
| BAC | | 2/8 | | | 0/3 | | | 0/2 | | | 0/1 | | |  | | | 2/2 | | |
| SEPSIS | | 14/9 | | | 2/1 | | | 1/0 | | | 1/1 | | |  | | | 10/7 | | |
| PUO/PUO-HO | | 5/3 | | | 1/0 | | | 4/1 | | | 0/2 | | |  | | |  | | |
| FN | | 5/9 | | | 1/0 | | | 3/3 | | | 1/6 | | |  | | |  | | |

Abbreviations: AICU, adult intensive care unit; AMW, adult medical ward; ASW, adult surgical ward; BAC, bacteremia; CAI, community-acquired infection; FN, febrile neutropenia; GNMW, general neonatal medical ward; HAI, healthcare-associated infection; HO-AMW, hematology-oncology AMW; NICU, neonatal intensive care unit; P-AMW, pneumology-AMW; PICU, pediatric intensive care unit; PMW, pediatric medical ward; PSW, pediatric surgical ward; PUO, pyrexia of unknown origin; PUO-HO, pyrexia of unknown origin in non-neutropenic hematology-oncology patient; T-AMW, transplant-AMW; UNK, unknown whether CAI or HAI.

**Supplementary Material Table S2.** Antimicrobial Prevalence by Medical Prophylaxis Site for Adult, Pediatric and Neonatal Wards

| **Adult** |  | **Overall** | | **AMW** | | **HO-AMW** | | **T-AMW** | | **P-AMW** | | **ASW** | **AICU** |
| --- | --- | --- | --- | --- | --- | --- | --- | --- | --- | --- | --- | --- | --- |
|  | **Total number of antimicrobials prescribed for medical prophylaxis** | 203 | | 80 | | 20 | | 55 | | 10 | | 20 | 18 |
|  | **Prophylaxis Site** |  | |  | |  | |  | |  | |  |  |
|  | Ear, Nose, Throat | 1 | |  | |  | |  | |  | | 1 |  |
|  | Respiratory Tract | 37 | | 19 | | 2 | |  | | 9 | | 1 | 6 |
|  | Cardiovascular System | 1 | |  | |  | |  | |  | | 1 |  |
|  | Gastrointestinal Tract | 15 | | 5 | |  | |  | |  | | 5 | 5 |
|  | Bone and Joint | 5 | | 3 | |  | |  | |  | | 2 |  |
|  | Urinary Tract | 5 | | 3 | |  | |  | |  | |  | 2 |
|  | Obstetrics or Gynecological | 5 | | 5 | |  | |  | |  | |  |  |
|  | General medical prophylaxis without targeting a specific site | 120 | | 41 | | 18 | | 45 | | 1 | | 10 | 5 |
|  | Bacteremia with no clear anatomic site and no shock | 2 | |  | |  | | 2 | |  | |  |  |
|  | Fever in a neutropenic patient | 12 | | 4 | |  | | 8 | |  | |  |  |
| **Pediatric and Neonatal** |  | | **Overall** | | **PMW and GNMW** | | **HO-PMW** | | **T-PMW** | | **PSW** | | **PICU and NICU** |
|  | **Total number of antimicrobials prescribed for medical prophylaxis** | | 85 | | 11 | | 33 | | 22 | | 4 | | 15 |
|  | **Prophylaxis Site** | |  | |  | |  | |  | |  | |  |
|  | Ear, Nose, Throat | | 1 | |  | |  | | 1 | |  | |  |
|  | Respiratory Tract | | 27 | | 1 | | 19 | | 2 | | 3 | | 2 |
|  | Gastrointestinal Tract | | 3 | | 2 | |  | | 1 | |  | |  |
|  | Urinary Tract | | 6 | | 2 | |  | | 2 | |  | | 2 |
|  | General medical prophylaxis without targeting a specific site | | 40 | | 6 | | 14 | | 17 | |  | | 3 |
|  | Medical prophylaxis for maternal risk factors | | 2 | |  | |  | |  | |  | | 2 |
|  | Medical prophylaxis for newborn risk factors | | 6 | |  | |  | |  | |  | | 6 |

Abbreviations: AICU, adult intensive care unit; AMW, adult medical ward; ASW, adult surgical ward; GNMW, general neonatal medical ward; HO-AMW, hematology-oncology AMW; NICU, neonatal intensive care unit; P-AMW, pneumology-AMW; PICU, pediatric intensive care unit; PMW, pediatric medical ward; PSW, pediatric surgical ward; T-AMW, transplant-AMW.

**Supplementary Material Table S3.** Antimicrobial Prevalence by Surgical Prophylaxis Site for Adult, Pediatric and Neonatal Wards

| **Adult** |  | **Overall** | | **AMW** | | **HO-AMW** | **T-AMW** | **P-AMW** | | **ASW** | **AICU** |
| --- | --- | --- | --- | --- | --- | --- | --- | --- | --- | --- | --- |
|  | **Total number of antimicrobials prescribed for surgical prophylaxis** | 137 | | 20 | | 0 | 2 | 0 | | 83 | 32 |
|  | **Surgical Site** |  | |  | |  |  |  | |  |  |
|  | Central Nervous System | 7 | |  | |  |  |  | | 3 | 4 |
|  | Ear, Nose, Throat | 4 | |  | |  |  |  | | 4 |  |
|  | Cardiovascular System | 37 | | 1 | |  |  |  | | 14 | 22 |
|  | Gastrointestinal Tract | 30 | |  | |  | 2 |  | | 24 | 4 |
|  | Skin and Soft Tissue | 6 | | 5 | |  |  |  | |  | 1 |
|  | Bone and Joint | 29 | |  | |  |  |  | | 28 | 1 |
|  | Urinary Tract | 10 | | 1 | |  |  |  | | 9 |  |
|  | Obstetric or Gynecological | 14 | | 13 | |  |  |  | | 1 |  |
| **Pediatric and Neonatal** |  | | **Overall** | | **PMW and GNMW** | | **HO-PMW** | | **T-PMW** | **PSW** | **PICU and NICU** |
|  | **Total number of antimicrobials prescribed for surgical prophylaxis** | | 33 | | 5 | | 2 | | 0 | 17 | 9 |
|  | **Surgical Site** | |  | |  | |  | |  |  |  |
|  | Central Nervous System | | 3 | |  | |  | |  | 3 |  |
|  | Ear, Nose, Throat | | 5 | | 3 | | 1 | |  | 1 |  |
|  | Cardiovascular System | | 11 | | 2 | |  | |  | 2 | 7 |
|  | Gastrointestinal System | | 6 | |  | |  | |  | 5 | 1 |
|  | Bone and Joint | | 7 | |  | | 1 | |  | 6 |  |
|  | Urinary Tract | | 1 | |  | |  | |  |  | 1 |

Abbreviations: AICU, adult intensive care unit; AMW, adult medical ward; ASW, adult surgical ward; GNMW, general neonatal medical ward; HO-AMW, hematology-oncology AMW; NICU, neonatal intensive care unit; P-AMW, pneumology-AMW; PICU, pediatric intensive care unit; PMW, pediatric medical ward; PSW, pediatric surgical ward; T-AMW, transplant-AMW.
